# PIMS (Positioning In Macular hole Surgery) trial – a multicentre interventional comparative randomised controlled clinical trial comparing face-down positioning, with an inactive face-forward position on the outcome of surgery for large macular holes: study protocol for a randomised controlled trial

**DOI:** 10.1186/s13063-015-1048-8

**Published:** 2015-11-17

**Authors:** Saruban Pasu, Catey Bunce, Richard Hooper, Ann Thomson, James Bainbridge

**Affiliations:** Moorfields Eye Hospital NHS Foundation Trust, City Road, EC1V 2PD London, UK; NIHR Biomedical Research Centre (BRC) at Moorfields Eye Hospital NHS Foundation Trust and UCL Institute of Ophthalmology, London, UK; Pragmatic Clinical Trial Unit, Blizard Institute Barts and The London School of Medicine and Dentistry, 4 Newark Street, London, E1 2AT UK

## Abstract

**Background:**

Idiopathic macular holes are an important cause of blindness. They have an annual incidence of 8 per 100,000 individuals, and prevalence of 0.2 to 3.3 per 1000 individuals with visual impairment. The condition occurs more frequently in adults aged 75 years or older. Macular holes can be repaired by surgery in which the causative tractional forces in the eye are released and a temporary bubble of gas is injected. To promote successful hole closure individuals may be advised to maintain a face-down position for up to 10 days following surgery. The aim of this study is to determine whether advice to position face-down improves the surgical success rate of closure of large (>400 μm) macular holes, and thereby reduces the need for further surgery.

**Methods/Design:**

This will be a multicentre interventional, comparative randomised controlled clinical trial comparing face-down positioning with face-forward positioning.

At the conclusion of standardised surgery across all sites, participants still eligible for inclusion will be allocated randomly 1:1 to 1 of the 2 treatment arms stratified by site, using random permuted blocks of size 4 or 6 in equal proportions. We will recruit 192 participants having surgery for large macular holes (>400 μm); 96 in each of the 2 arms of the study. The primary objective is to determine the impact of face-down positioning on the likelihood of closure of large (≥400 μm) full-thickness macular holes following surgery.

**Discussion:**

This will be the first multicentre randomised control trial to investigate the value of face-down positioning following macular hole standardised surgery.

**Trial registration:**

UK CRN: 17966 (date of registration 26 November 2014).

**Electronic supplementary material:**

The online version of this article (doi:10.1186/s13063-015-1048-8) contains supplementary material, which is available to authorized users.

## Introduction

### Background

Idiopathic macular holes are an important cause of blindness. They have an annual incidence of 8 per 100,000 individuals [1], and a prevalence of 0.2 [2] to 3.3 [3] per 1000 individuals with visual impairment. The condition occurs more frequently in adults aged 75 years or older [2]. Macular holes can have a devastating impact on quality of life and independence but can be repaired by surgery in which the causative tractional forces in the eye are released and a temporary bubble of gas is injected. To promote successful hole closure individuals may be advised to maintain a face-down position for up to 10 days following surgery. However, face-down positioning can be arduous [4] and associated with significant adverse effects [5], and evidence of its value is lacking. A recent Cochrane review [6] highlighted the need for an appropriately designed, adequately powered randomised controlled clinical trial to determine with confidence its value. The review reported that estimated effects were in favour of positioning but differences were not statistically significant for smaller macular holes. It concluded that face-down positioning may improve the likelihood of successful surgery for large macular holes (400 μm or greater in diameter) and highlighted the need for an appropriately designed, adequately powered randomised controlled clinical trial to determine with confidence the value of advice to position face-down.

### Rationale

The benefit of face-down positioning to the success of surgery is unproven; evidence to guide individuals on its impact is lacking and advice offered by clinicians varies widely. The current lack of evidence with which to guide patients has led to a lack of consensus among clinicians and wide variation in clinical practice.

To address the limitations in the evidence we will perform a trial to determine whether advice to position face-down, as opposed to face-forward, improves the probability of macular hole closure at 3 months after surgery, and so reduces the need for further surgery. We will test whether advice to position face-down results in a better outcome than an inactive face-forward position following surgery for large macular holes (≥400 μm).

A previous pilot randomised controlled trial (RCT) [7] has demonstrated the feasibility of this definitive RCT.

We have chosen to include only large macular holes (≥400 μm) because the available evidence suggests that face-down positioning is of more relevance to larger holes than for smaller macular holes [7–9]. In addition, the role of surgery in the management of smaller macular holes may be influenced in the future by the introduction of enzymatic vitreolysis techniques; intra-ocular injection of ocriplasmin can help induce closure in up to 40 % of smaller macular holes [10]. However, this approach is not designed for larger holes, the management of which is likely to involve surgery for the foreseeable future.

The proposed trial has been designed taking into account the views of individuals with macular holes and clinicians. The pilot study suggested a benefit of positioning following surgery for large macular holes. Previous pilot study subjects were invited to form a Patient Advisory Panel to advise on the design and methodology of this new RCT. Both patients and clinicians indicated that randomisation to either face-down positioning for as long as 10 days, or conversely to no positioning at all, would adversely affect recruitment to the trial because these alternatives are typically considered too arduous or would present too high a risk of non-closure, respectively. Instead, we have reached a consensus, based on the approach used by Guillaubey et al [8], on the alternatives of 5 days positioning either face-down or in an inactive face-forward position.

For the primary outcome we have chosen successful closure of the macular hole because, according to our Patient Advisory Panel, this is directly relevant to patients; hole closure is essential for a favourable impact on sight and determines whether further surgery is required. Although visual acuity is a relevant measure of functional outcome, its validity is limited to some extent in the short term by the confounding influence of secondary lens opacity and does not directly determine the need for further surgery.

We have chosen not to attempt to measure objectively the compliance of subjects with positioning. This is because self-reported compliance is of unknown reliability, and objective monitoring risks influencing behaviour artificially and unpredictably. Instead, we have taken the pragmatic approach to determine the value of the *advice* to patients regarding position. However, following the period of positioning we will ask subjects to say retrospectively how easy they found it to maintain their allocated position (on a scale of 0 (very difficult) to 10 (very easy). We will also assess their quality of life using the National Eye Institute Visual Function Questionnaire (VFQ-25).

The trial will benefit patients by providing reliable information on the value of positioning following surgery for large macular holes, thereby enabling them to make an appropriately informed choice about the management of their condition.

The research will benefit the National Health Service (NHS) because by determining the value of face-down positioning we can expect to improve the likelihood of prompt successful surgery and hence reduce the amount of additional resource required for further clinical management and associated costs.

### Objectives

The aim is to determine whether advice to position face-down improves the surgical success rate of closure of large (≥400 μm) macular holes, and thereby reduces the need for further surgery.

#### Primary objective

The primary objective is to determine the impact of face-down positioning on the likelihood of closure of large (≥400 μm) full-thickness macular holes following surgery.

#### Secondary objective

The secondary objective is to determine the impact of face-down positioning on sight, quality of life and wellbeing.

## Methods/Design

This will be a multicentre interventional, comparative randomised controlled clinical trial comparing face-down positioning with face-forward positioning.

We will recruit 192 participants having surgery for large macular holes (≥400 μm); 96 in each of the 2 arms of the study.

### Inclusion criteria

1. Presence of an idiopathic full-thickness macular hole, greater than or equal to 400 μm in diameter as measured by optical coherence tomography (OCT).
2. Patients electing to have surgery for a macular hole, with or without simultaneous phacoemulsification and intra-ocular lens implant.
3. Ability and willingness to position face-down or in an inactive face-forward position.

### Exclusion criteria

1. Age-related macular degeneration; glaucoma; diabetic retinopathy; retinal degeneration; amblyopia; previous vitrectomy surgery (refractive error, lens opacity and previous use of ocriplasmin are not exclusion criteria).
2. Traumatic macular hole.
3. History of visual loss suggesting macular hole duration longer than 12 months.
4. Presence of a retinal tear identified during surgery for which postoperative positioning is advised.

## Informed consent

Eligible candidates will be approached at their baseline visit by the clinical team, provided with information about the trial and invited to participate. They will be given time to consider their decision and the opportunity to ask questions. Investigators will ensure that information about equipoise is provided impartially so as to avoid potential bias by influencing compliance with advice to position. Should candidates elect to participate, informed consent will be obtained (see Additional file 1) at the time of listing for surgery. Candidates will understand that that although consenting to participate in the trial, they will be formally enrolled only if the inclusion and exclusion criteria are still met following surgery.

We will not be recruiting/consenting subjects who we feel lack capacity.

The surgery will involve vitrectomy (using instruments of any gauge), internal limiting membrane (ILM) peeling (with or without staining by a vital dye), fluid-air exchange and injection of octafluoropropane (C3F8) 14 % gas. Vitrectomy may be combined with phacoemulsification and intra-ocular lens implant.

Subjects will be randomised immediately following surgery, unless exclusion criteria are met during surgery.

### Intervention

The intervention is advice to adopt face-down positioning (see Additional file 2): subjects will be advised to maintain a face-down position for a total of at least 8 consecutive or non- consecutive hours a day for 5 days following surgery.

The comparator is advice to adopt face-forward positioning (see Additional file 3): subjects will be advised to maintain a face-forward position, inactive, for at least 8 consecutive or non-consecutive hours a day for 5 days following surgery.

Subjects in either group will be allowed a 15-minute break (every hour) from their allocated position. In the position-free-time we will advise subjects to avoid the face-up position.

Face-down or face-forward positioning will be advised during waking hours only, not during sleep. We will advise subjects to avoid the face-up position during sleep.

Investigators will explain positioning to candidates prior to surgery, providing written instructions with diagrams.

### Randomisation

Eligible candidates will be invited to participate and to sign the consent form prior to surgery.

At the conclusion of standardised surgery across all sites, participants still eligible for inclusion will be allocated randomly 1:1 to one of the 2 treatment arms (refer to the flow diagram in Additional file 4) stratified by site, using random permuted blocks of size 4 or 6 in equal proportions.

Randomisation will be performed using a secure bespoke online randomisation service implemented by the PCTU (Pragmatic Clinical Trials Unit). Each site will be provided with a unique log-in username and password to access the service. Online randomisation will be available 24 hours a day, 7 days a week apart from short periods of scheduled maintenance. Access to the service will be restricted to staff as delegated by the Principal Investigator (PI). They will input the participant ID and details required for randomisation, and then will be presented with immediate on-screen randomisation. The randomisation system will have built-in checks to check that (1) the participant ID is not a duplicate, and (2) the date of birth is within the eligible age set in the protocol.

Once the subject has been randomised, the enrolment of this subject will be documented on the enrolment log. An Email will be automatically generated to notify the Chief Investigator’s (CIs) team and PCTU of all participants randomised to the trial.

### Masking

Subjects and clinicians will be unmasked to treatment allocation.

Investigators assessing the primary endpoint by grading of OCT scans will be masked to treatment allocation. This will be achieved by electronic capture of OCT images which are presented anonymously to the grading clinicians. Each clinician will be masked to their colleagues grading. Postoperative hole closure will be determined according to the presence (open) or absence (closed) of any gap between the opposing edges of the hole. No measurement is involved. In the event of disagreement between clinicians, the opinion of a third clinician (also masked) will be sought.

### Follow-up

Subjects will attend for follow-up assessment as part of the trial at 3 months following surgery. Their surgical teams will manage their routine postoperative clinical care in the meantime.

The primary outcome, macular hole closure, will be determined by masked assessment of OCT scans acquired at 3 months.

### Unit of analysis

All ocular assessments relate to the study eye. In the event that a subject is having surgery for bilateral macular holes (which are not operated on simultaneously), the first eye to be operated on during the trial will be the study eye.

### Data collection

Table 1 shows the schedule of assessments.

**Table 1:** Schedule of assessments

Assessment	Preoperative	Surgery	3 months postoperative
Age	x		
Sex	x		
Ethnicity	x		
Laterality	x		
Duration of symptoms	x		
BCVA	x		x
Lens status: phakic/pseudophakic	x		x
Informed consent	x		
Surgery		x	
Randomisation		x	
Macular hole diameter on OCT	x		
Macular hole status (closed; open flat; open elevated)			x
QoL VFQ-25 questionnaire	x		x
Subject-reported experience of positioning			x
If primary repair of macular hole failed, was second operation performed/planned			x

*BCVA* best-corrected visual acuity, *OCT* optical coherence tomography, *QoL* quality of life, *VFQ-25* Visual Functional Questionnaire

Pre-operative data collection:

- Demographic data (age, sex, ethnicity)
- Laterality
- Duration of symptoms
- Best-corrected visual acuity (BCVA) measured using Snellen charts at a standard distance of 6 metres
- OCT with recording of minimal hole diameter (see Additional file 5)

### Outcome measures

#### Primary outcome

The primary outcome will be anatomical closure of the macular hole, determined at 3 months after surgery by masked assessment of OCT scans.

#### Secondary outcomes

- Further surgery for macular hole, performed or planned
- BCVA using standard Snellen chart at 6 metres
- Patient-reported experience of positioning at 3 months
- Patient-reported health and quality of life as assessed at baseline and at 3 months using the National Eye Institute Visual Function Questionnaire (VFQ-25)

### End of study definition

The end of the study will be at the final assessment of the final subject.

## Statistical considerations

### Sample size and power calculation

Clinical consensus is that face-down positioning would be recommended if there were a difference of 15 % in success rates. This is the smallest clinically relevant treatment difference that we wish to detect. Previous studies [8] indicate that successful closure of large macular holes without advice to position face-down is 80 %. A study with 86 patients per group has 85 % power to detect a difference between 80 % in the face-forward positioning arm and 95 % in the face-down positioning arm. With a 10 % loss to follow-up, we are aiming to recruit 96 patients in each arm.

### Analysis

Baseline characteristics will be tabulated in the two treatment arms.

Proportions of macular hole closures at 3 months will be compared between treatment arms using logistic regression adjusting for age and sex, with site as a random effect.

Visual acuity at 3 months will be compared using linear regression adjusting for age, sex, and baseline visual acuity, with site as a random effect. We will also adjust for surgery type in the logistic regression analysis. Questionnaire scores assessed at 3 months will be compared using linear regression adjusting for age and sex, with site as a random effect.

The numbers of participants who decline to participate, fail screening, or withdraw or are lost to follow-up will be recorded in a Consolidated Standards of Reporting Trials (CONSORT) flow-chart.

The analyses will be on an intention-to-treat basis, and every effort will be made to collect complete data. If any outcome data are missing we will analyse available subjects only (this is unbiased under a missing-at-random assumption where missingness depends only on variables adjusted for in the analysis), but we will also perform secondary analyses investigating the missing-at-random assumption and involving further baseline covariates if necessary.

OCT scans will be anonymised and sent to two independent retinal surgeons who will grade the macular hole as closed; open and flat (without a cuff of subretinal fluid), or open and elevated (with a cuff of subretinal fluid). The readers will be masked to the identity and treatment allocation of the subject. In the event of any disparity in grading, a third independent retinal surgeon, also masked to identity and treatment allocation, will arbitrate.

A formal statistical analysis plan will be signed off by the Trial Steering Committee prior to analysis.

### Safety considerations

Random allocation to the alternatives of face-down and face-forward positioning presents a possible safety issue because of the uncertainly over which is more effective, and the known adverse effects of prolonged face-down positioning. We have addressed these risks by advising a minimum of inactive face-forward positioning for all subjects, and only an 8-hour total minimum period of face-down positioning.

### Data handling and record keeping

- Confidentiality
  Information related to participants should be kept confidential and managed in accordance with the Data Protection Act, NHS Caldecott Principles, The Research Governance Framework for Health and Social Care, and the conditions of Research Ethics Committee (REC) approval.
- Record retention and archiving
  All study documents are to be retained for a period of 5 years following conclusion of the study. Following the submission of the end of study report the sponsor will arrange for archiving of the Trial Master File in accordance with the sponsor’s process. The sponsor will also notify the local PIs that the Investigator Site Files (ISFs) may be archived. The ISFs will be retained and archived at site in accordance with the Trusts’ procedures.
  Following the end of the retention period the sponsor will notify the PIs in writing that the required retention period has completed and that documents can be destroyed. A copy of the instruction to the Trust Archivist to destroy the ISF will be requested.

## Products, devices, techniques and tools

### Devices

Spectral domain ocular coherence tomography (SD-OCT) will be used at the various sites to determine the preoperative size of the macular hole, and whether surgery has been successful in terms of hole closure at 3 months.

The size of the macular hole is defined as its minimum horizontal diameter. This is its linear width measured using the OCT caliper function along a line that bisects the hole in the horizontal meridian and is parallel to the retinal pigment epithelium (see Additional file 5).

### Tools

The VFQ-25 is a reliable and valid 25-item version of the 51-item National Eye Institute Visual Function Questionnaire (NEI-VFQ).

## Safety reporting

### Adverse event (AE)

Safety reporting will adhere to the sponsor’s standard operating procedures. If the AE is not defined as serious, it will be recorded in the study file and the participant will be followed-up by the research team. The AE will be documented in the participants’ medical notes.

### Serious adverse event (SAE)

A SAE is defined as an untoward occurrence that:

- Results in death;
- Is life-threatening;
- Requires hospitalisation or prolongation of existing hospitalisation;
- Results in persistent or significant disability or incapacity;
- Consists of a congenital anomaly or birth defect; or
- Is otherwise considered medically significant by the investigator.

An SAE occurring to a research participant will be reported to the main REC where in the opinion of the CI the event was:

- Related – that is, it resulted from administration of any of the research procedures, and
- Unexpected – that is, the type of event is not listed in the protocol as an expected occurrence.

Expected AEs include the following:

- Ocular discomfort; epiphora; periocular swelling; diplopia; ptosis
- Subconjunctival or intra-ocular hemorrhage
- Corneal abrasion
- Retinal or choroidal tear or detachment
- Wound leak
- Ocular hypotony or raised intra-ocular pressure/glaucoma
- Overfill or underfill of intra-ocular gas tamponade
- Intra-ocular or extra-ocular inflammation or infection
- Intra-ocular neovascularisation
- Lens opacity, subluxation or dislocation of lens or lens implant
- Persistent or recurrent macular hole
- Visual field defect or other disturbance of sight
- Discomfort of joints, neck, back or limbs

SAEs that are considered to be ‘related’ and ‘unexpected’ are to be reported to the sponsor within 24 hours of learning of the event using the following Email address: pharmacovigilance@moorfields.nhs.uk and to the main REC within 15 days, in line with the required timeframe. SAEs will be documented in the participants’ medical notes and the Case Report Form (CRF).

### Urgent safety measures

The CI may take urgent safety measures to ensure the safety and protection of the clinical trial subjects from any immediate hazard to their health and safety. The measures should be taken immediately. In this instance, the approval of the REC prior to implementing these safety measures is not required. However, it is the responsibility of the CI to inform the sponsor and main REC (via telephone) of this event immediately.

The CI has an obligation to inform both the main REC in writing within 3 days, in the form of a substantial amendment. The sponsor must be sent a copy of the correspondence with regards to this matter.

### Annual safety reporting

The CI will send the Annual Progress Report to the main REC using the National Research Ethics Service (NRES) template (the anniversary date is the date on the ‘favourable opinion’ letter from the REC) and to the sponsor.

### Overview of the safety reporting responsibilities

The CI has the overall safety oversight responsibility and will ensure that safety monitoring and reporting is conducted in accordance with the sponsor’s requirements.

### Monitoring and auditing

The study will be monitored in line with the study monitoring plan, written by the PCTU quality assurance (QA) manager and agreed by the study sponsor. The PCTU has provisionally identified this study as being medium risk.

### Trial organisation

#### Trial management committee

James Bainbridge: Chief Investigator, Moorfields Eye Hospital, London, UK

Saruban Pasu: Co-investigator, NIHR Biomedical Research Centre (BRC) at Moorfields Eye Hospital NHS Foundation Trust and UCL Institute of Ophthalmology, London, UK

Catey Bunce: Co-investigator and statistician, Moorfields Eye Hospital, London, UK

Ann Thomson: Senior Trial Manager, Pragmatic Clinical Trials Unit, London, UK

Irene Simmonds: Trial Co-ordinator, Pragmatic Clinical Trials Unit, London, UK

Richard Hooper: Statistician, Pragmatic Clinical Trials Unit, London, UK

Mike Waring: Data Manager, Pragmatic Clinical Trials Unit, London, UK

#### Trial steering committee

Noemi Lois: Consultant Ophthalmic Surgeon, Belfast Health and Social Care Trust, Belfast, Northern Ireland, UK.

Simon Skene: Senior Statistician, University College London, London, UK.

Roy Smith: Lay Person, UK.

This team will also perform the duties of a data management committee.

### Finance and funding

The trial is funded by the National Institute for Health Research Health Technology Assessment (NIHR HTA) Research for Patient Benefit scheme.

### Indemnity

The sponsor is Moorfields Eye Hospital (MEH) NHS Foundation Trust, which participates in the Clinical Negligence Scheme for Trusts (CNST), run by the NHS Litigation Authority, which pools the risk of clinical negligence claims. NHS indemnity (for negligent harm) will cover MEH employees, both substantive and honorary, who are working in the course of their NHS employment and in respect of conducting research projects, which must have received NHS Permission. MEH will not accept liability for any activity that has not been properly registered and Trust approved.

### Ethics

Random allocation to the alternatives of face-down or face-forward positioning presents a possible ethical issue because of the uncertainty over which is safer and more effective. In particular, there is some concern that individuals with large holes randomised to non-positioning may be less likely to benefit from hole-closure. We have addressed this concern by ensuring that subjects not allocated to face-down positioning are advised to maintain an inactive face-forward position, which has been an acceptable standard for previous trials. If the results demonstrate that positioning face-down is more effective than positioning inactively face-forward we can conclude that it is also likely to be more effective than not positioning at all.

Applications to the UK’s main REC (NRES Committee London – Westminster) and the local Moorfields Research Management Committee have received favourable opinion (REC reference 14/LO/2061).

We will perform the study in accordance with the ethical principles in the Research Governance Framework for Health and Social Care, Second Edition, 2005 and its subsequent amendments.

### Dissemination of research findings

The results will be disseminated at clinical meetings, and by publication in a peer-reviewed journal.

## Discussion

The research team combines the strengths of experienced eye specialists with the expertise of the PCTU, and the active involvement of patients to ensure that the trial addresses their needs. The trial will benefit patients by providing reliable information on the value of posturing following surgery for large macular holes, thereby enabling them to make an appropriately informed choice about the management of their condition.

## Trial status

The authors confirm that the trial will start recruiting from May 2015 onwards.

## Additional files

Additional file 1:
**Consent form.** (DOCX 38 kb) Additional file 2:
**Examples of face-down seated and face-down lying.** (DOCX 446 kb) Additional file 3:
**Examples of face-forward reading and face-forward watching TV.** (DOCX 414 kb) Additional file 4:
**Flow diagram of PIMS trial.** (DOCX 39 kb) Additional file 5:
**OCT image of macular hole. **(DOCX 627 kb)

## Abbreviations

AE: adverse event
BRC: Biomedical Research Centre
BCVA: best-corrected visual acuity
CNST: Clinical Negligence Scheme for Trusts
CONSORT: Consolidated Standards of Reporting Trials
CI: Chief Investigator
CRF: Case Report Form
HTA: Health Technology Assessment
ILM: internal limiting membrane
IFS: Investigator Site Files
NIHR: National Institute for Health Research
NHS: National Health Service
MEH: Moorfields Eye Hospital
NEI: National Eye Institute
NRES: National Research Ethics Service
OCT: optical coherence tomography
PCTU: Pragmatic Clinical Trials Unit
PI: Principal Investigator
QA: quality assurance
RCT: randomised controlled trial
REC: Research Ethics Committee
SAE: serious adverse event
SD-OCT: spectral domain ocular coherence tomography
VFQ: Visual Functional Questionnaire
