# Supplementary material for: PIMS (Positioning In Macular hole Surgery) trial – a multicentre interventional comparative randomised controlled clinical trial comparing face-down positioning, with an inactive face-forward position on the outcome of surgery for large macular holes: study protocol for a randomised controlled trial
Source: Trials. 2015 Nov 17;16:527. doi: 10.1186/s13063-015-1048-8 (PMC4650938; doi:10.1186/s13063-015-1048-8)
Supplement: Additional file 1: — Consent form. (DOCX 38 kb) [file 13063_2015_1048_MOESM1_ESM.docx]

**Additional file 1**

**PATIENT ID NUMBER:**

| **PARTICIPANT CONSENT FORM**  **PIMS trial: Positioning in Macular Hole Surgery Trial** |
| --- |

**Please initial box to indicate agreement**

| 1. | I confirm I have read and understood Patient Information Sheet v1.6 Feb 5th Jan 2015 for the above study. I have had the opportunity to consider the information, ask questions and these questions have been answered satisfactorily. |  |
| --- | --- | --- |
| 2. | I understand that my participation is voluntary and that I am free to withdraw at any time, without giving any reason, without my medical care or legal rights being affected. |  |
| 3. | I understand that relevant sections of my medical notes and data collected during the study may be looked at by responsible individuals from the study team, regulatory authorities or by individuals delegated by the Sponsor, Moorfields Eye Hospital NHS Foundation Trust, where it is relevant to the research. I give permission for these individuals to have access to my records. |  |
| 4. | I agree to my GP being informed of my participation in the study. |  |
| 5. | I agree to the clinical data generated by this study being used for other research purposes in the future, after being appropriately anonymised. |  |
| 6. | I agree to take part in the above study. |  |

You will be provided with a signed copy of this consent form.

Print name of participant Signature Date

________________ ___________ _______________

Print name of person

taking consent Signature Date

**_________________ ______________ ________________**
