# Supplementary figures and images for: PIMS (Positioning In Macular hole Surgery) trial – a multicentre interventional comparative randomised controlled clinical trial comparing face-down positioning, with an inactive face-forward position on the outcome of surgery for large macular holes: study protocol for a randomised controlled trial
Source: Trials. 2015 Nov 17;16:527. doi: 10.1186/s13063-015-1048-8 (PMC4650938; doi:10.1186/s13063-015-1048-8)

**Additional file 2:**

**FACE-DOWN SEATED**

**
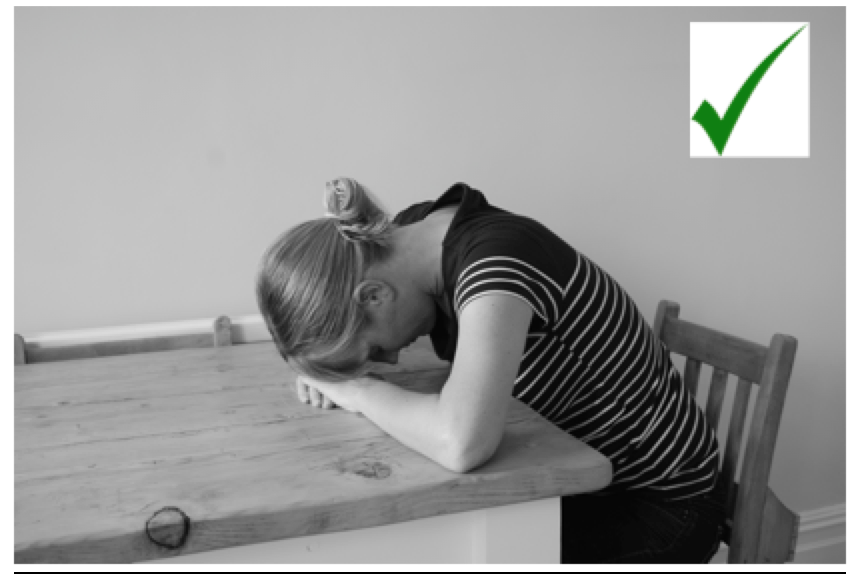
**

**FACE-DOWN LYING**

**
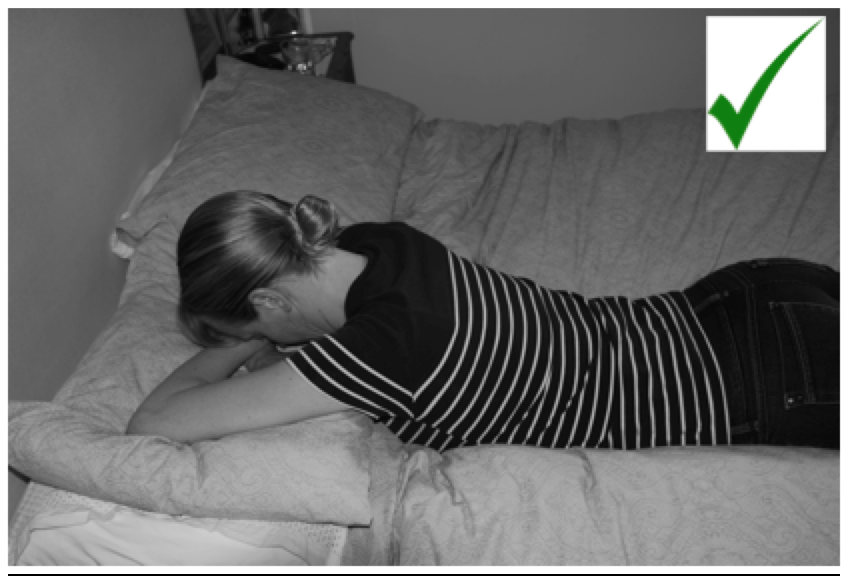
**

Supplement: Additional file 2: — Examples of face-down seated and face-down lying. (DOCX 446 kb) [file 13063_2015_1048_MOESM2_ESM.docx]

**Additional file 3:**

**FACE-FORWARD READING**

**
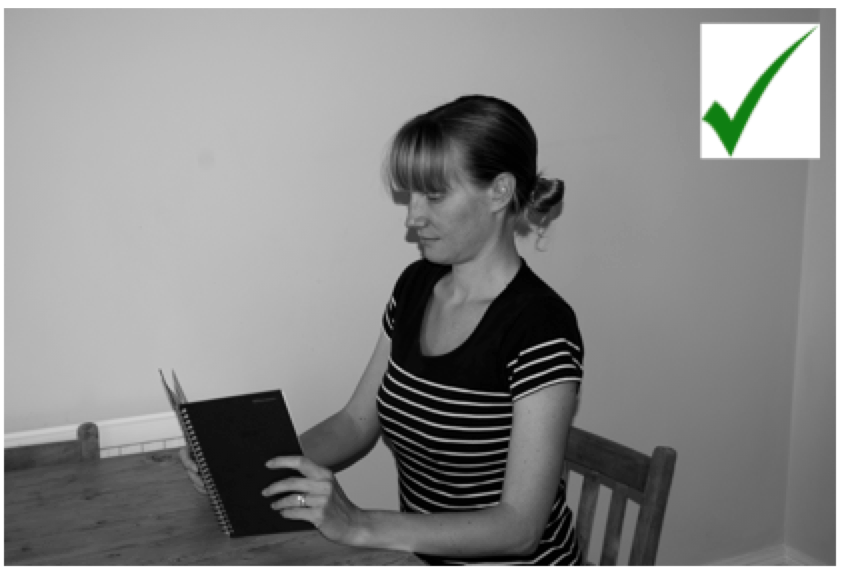
**

**FACE-FORWARD WATCHING TV**

**
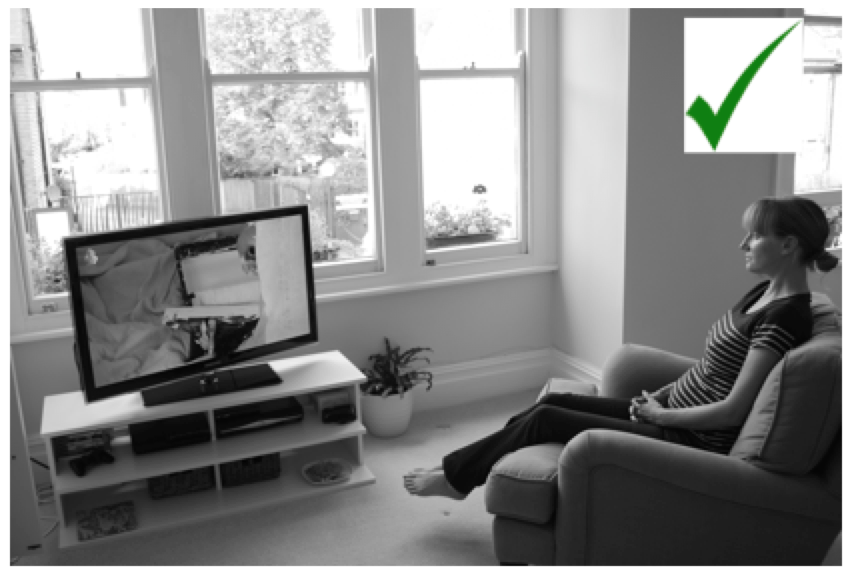
**

Supplement: Additional file 3: — Examples of face-forward reading and face-forward watching TV. (DOCX 414 kb) [file 13063_2015_1048_MOESM3_ESM.docx]
