# Supplementary material for: PIMS (Positioning In Macular hole Surgery) trial – a multicentre interventional comparative randomised controlled clinical trial comparing face-down positioning, with an inactive face-forward position on the outcome of surgery for large macular holes: study protocol for a randomised controlled trial
Source: Trials. 2015 Nov 17;16:527. doi: 10.1186/s13063-015-1048-8 (PMC4650938; doi:10.1186/s13063-015-1048-8)
Supplement: Additional file 4: — Flow diagram of PIMS trial. (DOCX 39 kb) [file 13063_2015_1048_MOESM4_ESM.docx]

**Additional file 4:**

**FLOW DIAGRAM OF PIMS TRIAL**

**Eligible candidate consents to participate**

**Baseline VFQ25 Questionnaire**

**Surgery performed**

**Candidates no longer eligible are excluded**

**Candidates still eligible are allocated to treatment by online randomisation**

**Face-forward**

**Face-down**

**Review at:**

**3 Months; hole status on OCT; VA; VFQ25 Questionnaire; subject experience**
