# Supplementary material for: PIMS (Positioning In Macular hole Surgery) trial – a multicentre interventional comparative randomised controlled clinical trial comparing face-down positioning, with an inactive face-forward position on the outcome of surgery for large macular holes: study protocol for a randomised controlled trial
Source: Trials. 2015 Nov 17;16:527. doi: 10.1186/s13063-015-1048-8 (PMC4650938; doi:10.1186/s13063-015-1048-8)
Supplement: Additional file 5: — OCT image of macular hole. (DOCX 627 kb) [file 13063_2015_1048_MOESM5_ESM.docx]

**Additional file 5:**

**OCT IMAGE OF MACULAR HOLE**


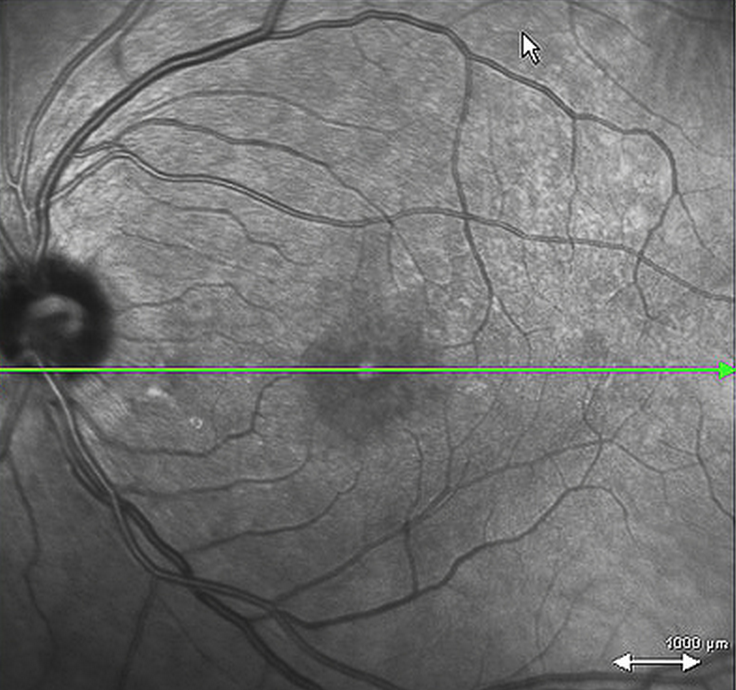


**
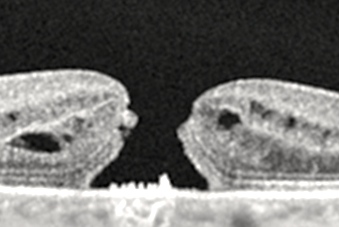
**

A

**A** = Minimum hole diameter

This is its linear width measured using the OCT caliper function along a line that bisects the hole in the horizontal meridian and is parallel to the retinal pigment epithelium.
